# Is there a special mechanism behind the changes in somatic cell and polymorphonuclear leukocyte counts, and composition of milk after a single prolonged milking interval in cows?

**DOI:** 10.1186/1751-0147-51-4

**Published:** 2009-01-15

**Authors:** Branislav Lakic, Ewa Wredle, Kerstin Svennersten-Sjaunja, Karin Östensson

**Affiliations:** 1 Department of Clinical Sciences, Division of Reproduction, Faculty of Veterinary Medicine and Animal Science, Swedish University of Agricultural Sciences, PO Box 7054, SE-750 07 Uppsala, Sweden; 2 Department of Animal Nutrition and Management, Swedish University of Agricultural Sciences (SLU), Uppsala, Sweden

## Abstract

**Background:**

A single prolonged milking interval (PMI) e.g. after a technical stop in an automated milking system is of concern for the producer since it is associated with a short-lasting increase in milk somatic cell count (SCC), which is a major quality criterion used at the dairy plants. The content of polymorphonuclear leukocytes (PMN) and how the milk quality is influenced has not been much investigated. The SCC peak occurs without any obvious antigen challenge, possibly indicating a different leukocyte attraction mechanism after a PMI than we see during mastitis.

**Methods:**

Composite cow milk samples were taken at the milkings twice daily during 7 days before and 5 days after a PMI of 24 h. Milk was analyzed for SCC, PMN, fat, protein and lactose, and at some occasions also casein and free fatty acids (FFA).

**Results:**

During the PMI the proportion of milk PMN increased sharply in spite of marginally increased SCC. The peak SCC was not observed until the second milking after the PMI, in the afternoon day 1. However, the peak SCC value in *morning *milk did not occur until one day later, concomitantly with a *decrease *in the proportion of PMN. After declining, SCC still remained elevated while PMN proportion was decreased throughout the study as was also the milk yield, after the first accumulation of milk during the PMI. Milk composition was changed the day after the PMI, (increased fat and protein content; decreased lactose, whey protein and FFA content) but the changes in the following days were not consistent except for lactose that remained decreased the rest of the study.

**Conclusion:**

The PMI resulted in increased SCC and proportion of PMN. Additionally, it gave rise to minor alterations in the milk composition in the following milkings but no adverse effect on milk quality was observed. The recruitment of PMN, which was further enhanced the first day *after *the PMI, appeared to be independent of milk volume or accumulation of milk per se. Hence, we suggest that there is a special immunophysiological/chemoattractant background to the increased migration of leukocytes into the milk compartment observed during and after the PMI.

## Background

A technical stop in an automated milking system (AMS) results in a prolonged milking interval (PMI) which for many cows may be fairly pronounced. Intervals of up to 24 hours have been observed (personal communication, Gunnar Pettersson, research manager, Kungsängen Research Centre, SLU, ). It has been noticed that many cows show a short-lasting increase in milk somatic cell count/ml (SCC) shortly after the stop [1]. It is well known that the SCC, besides inflammation, is influenced by several physiological and management factors [2], e.g. milking frequency, and it is reasonable to assume that the SCC peaks after a stop in an AMS are related to changed conditions in the udder due to the PMI. The length of the milking interval, if the same length is applied repeatedly during a period of time, has previously been shown to affect the SCC. Milking once a day increases the SCC [3,4] and very short (3 h) intervals have the same effect [5]. The short-lasting SCC peaks after a single PMI has, to our knowledge, been sparsely studied.

The majority of cells in bovine milk are leukocytes. The increase in milk SCC during mastitis is mainly due to enhanced recruitment of polymorphonuclear leukocytes (PMN) to the udder and milk as a result of chemotactic agents released during an inflammatory reaction. This leads to an increased proportion of PMN in the milk (see e.g. [6]) which has been shown to be a more sensitive inflammatory indicator than the total SCC [7-9]. When the SCC is influenced by other factors than mastitis, an increased SCC is also generally associated with increased proportion of PMN, in both individual cow and herd milk [7,9-11]. Accordingly, it has been shown that once-daily milking on a regular basis results in increased proportion of PMN along with the increased SCC [3] while, notably, one omitted milking has been reported *not *to influence the proportion of PMN [12] although the SCC is affected, possibly indicating a different underlying mechanism.

A substantial amount of leukocytes (monocytes/macrophages) is present in bovine milk also under healthy conditions (see e.g. [13]) although lower than during inflammation. Regulation of normal cell traffic and cell turn over in the udder is not well mapped. It can be speculated that an increase in milk SCC could be attributable mainly to increased numbers of other kind of leukocytes than PMN as a result of disturbed physiological cell traffic, which would indicate another inflammatory leukocyte attraction mechanism than usually seen during mastitis. It might also be possible that an increased proportion of PMN may be present in the milk without the total SCC being elevated. Particularly, the short lasting peaks where the SCC returns to normal spontaneously, within a day or even sooner, may be suspected to have a special underlying mechanism. Thus, by investigating the milk differential leukocyte count important information for understanding the background to the SCC changes can be gained.

Milking frequency (MF) has been shown to influence not only the cell content of milk but also the milk composition and yield. Milking cows, *regularly*, just once a day appears to result in reduced milk yield and lactose content, compared to milking two times per day, while fat and protein content increase (see e.g. [14]). The changes in composition seem to be common for both short- and long-term studies. However, how a *single *PMI influences milk composition and milk quality is not well documented. Increased SCC is, in general, associated with changes in the quantity, quality and composition of milk [15]. Besides the effect on the milk synthesis, elevated SCC has a direct negative effect on milk quality and shelf life [16]. In the milk quality control at the dairy, milk SCC has therefore been included as an important parameter and elevated SCC is often used as a basis for reduced payment to the farmer. The changes are most pronounced during clinical mastitis with lower yield and content of fat, casein and lactose [2,17]. Except for fat, such alterations with lower magnitudes have, however, been observed already when the SCC is moderately increased, and even during short-lasting periods [18,19].

The characteristics of the milk SCC peaks observed after a single PMI and how the various milk constituents may be affected during these peaks have, to our knowledge, not been studied. More information about the short-lasting, spontaneously declining SCC alterations would improve the knowledge about the cell traffic in the bovine mammary gland. Additionally, since the SCC peaks may influence the herd milk SCC which is used as a quality indicator of the milk delivered from the farm, it is also a matter of practical concern for the farmer, and the possible effect on milk quality and composition ought to be clarified.

The aim of the present study was to investigate the occurrence and pattern of episodes of elevated SCC in individual cows with low SCC after a single PMI and to clarify if the increased SCC is due to an enhanced recruitment of PMN. The aim was further to examine how the various milk components are influenced and if the milk quality is impaired in connection with short-lasting SCC peaks with this background.

## Methods

### Animals

The study was conducted at Kungsängen Research Centre, Swedish University of Agricultural Sciences (SLU), Uppsala, Sweden. Twenty-nine Swedish Red (SRB) cows were included in the experiment. Most of the cows were in mid lactation and in lactation number 1 or 2. The herd is comparable to an average Swedish dairy herd regarding lactation stage and age distribution among the cows.

All cows were free from clinical signs of mastitis and other health disturbances before the start of the study and had a cow composite milk SCC < 100 000. The cows were kept indoors in a tied up system. They were fed 4 times daily with silage and concentrate according to the Swedish recommendation. Milking was performed twice daily at 6.30 and 15.30 with a Duovac milking machine system (DeLaval, Tumba, Sweden). The average daily milk yield per cow in the herd according to the Swedish milk recording before the start of the study was 24.8 kg ECM (energy corrected milk). The study was approved by the Uppsala Local Ethics Committee.

### Sampling and experimental design

The duration of the study was 12 days in total during which the cows were exposed to a single PMI of 24 h at day 0 by excluding the afternoon milking. In the rest of the study milking was performed twice daily. Samples of approximately 40 ml of composite cow milk were taken at every milking at day -7, -3, -2, -1, 0, +1, +2, +3, +4 and +5. Additionally, samples of approximately 80 ml of composite cow milk for analysis of casein and FFA were collected in the afternoon milkings, at days -1 and 1 from all cows. At days 3 and 5 casein and FFA was analysed in milk only from cows which during day 1 had a pronounced reaction with SCC that was increased at least 2-fold compared to the afternoon sampling day -1, up to a total SCC value of at least 100 × 10^3^/ml. This has been suggested to be the plausible limit between inflamed and non-inflamed udders in cows [20,21]. Each sample was split up in aliquots for the different analyses and stored in 4°C until analyzed. Milk yield was measured at each milking by true test equipment.

### Milk analyses

The SCC was analyzed in fresh milk with no additives by fluorescence-based electronic cell count (Fossomatic 5000, A/S N. Foss Electric, Denmark) in a routine diagnostic laboratory. PMN were counted in 20 micro litre of milk in light microscope after Newman staining according to a modified version of the IDF standard (IDF 148-1/ISO/DIS 13366-1). The content of fat, protein and lactose, respectively, was analyzed by spectroscopic mid infrared technique (MIR; MilcoScan FT 120 A/S N. Foss Electric, Hillerød, Denmark). Samples intended for casein analysis were stored in 4°C in cans with a preservative (bronopole) until analyzed (Arla Foods analysis regulation 2000.004, 200001210). The proportion of casein was calculated from the whey protein and total protein proportions, using a rennet casein method. In short, 60 μl calcium chloride (48% w/v) was added to 40 ml of milk sample and incubated at 40°C in water bath. When the temperature reached 40°C, 200 μl rennet (180 ± 10 international milk clothing units) were added and samples were mixed and left to coagulate 15–20 min. The curd was cut into small cubes and then filtered (42 μm) to receive the whey protein fraction which was analyzed with mid infrared spectroscopy. FFA content was analysed by the Auto analyzer II method [22] after the samples had been stored in 4°C for 24 h. All other analyses and preparations of smears for PMN counting were performed within 6 h.

### Statistical analysis

Data of milk SCC, PMN, fat, protein and lactose were used in the statistical calculations. Additionally, to get a measure where a possible effect of the different length of day (9 h) and night (15 h) milking intervals, respectively, and of different milk volume (dilution/concentration effect) could be minimized, the average output per time unit (output/hr) since the previous milking of each parameter was calculated and tested. The output/hr was calculated by dividing the content in total milk volume (total milk content) for each parameter measured at each milking occasion with the number of hours that had elapsed since the immediately preceding milking. However, for casein and FFA which were analyzed in afternoon milk only, the total output per milking was used. The data were analyzed using the Mixed procedure with repeated measure ANOVA (Analysis of Variance) in SAS 9.1 (SAS Institute, Cary, NC, USA, 2002). To obtain normal distribution, the data on SCC were transformed to 10 logarithmic values before the analysis. The following model was used:

The model was for observed value of cow i at day t:

y_it _= μ + c_i _+ α_t _+ ε_it_

Where μ = overall mean, c_i_= random effect of the cow, α_t _= effect of sampling day t, ε_it _= random error. The error ε_it _and ε_ijt _corresponding to day t and μ are assumed to follow autoregressive dependence with correlation λ^/t-α/^. The covariance structure is accomplished by specified SP(POW) in the SAS program.

Before establishing the final statistical model grouping into two groups, according to milk yield and SCC, respectively, prior to the PMI was tested. Further, the influences of days in milk and lactation number on the different variables were tested.

The model used for testing the effect of group i at day t:

y_ijt _= μ + γ_i _+ c_ij _+ α_t _+ (γα)_it _+ ε_ijt_

Where γ_i _= effect of group and (γα)_it _is the interaction of group and day. The other effects are defined as in model 1.

After analysis it was revealed that none of mentioned parameters had any effect on the different variables, and that only days in milk, had a significant effect on milk composition, only. Thus, the final model included the parameter days in milk. The data are presented as least square means (LSM) with its standard error. After significant F-test (p < 0.05) least square means were compared in pair wise t-tests at the 5% level. The baseline value, with which values obtained after the PMI were compared, was calculated as the mean of the values for each parameter in all samples collected before the PMI.

## Results

**Figure 1:**
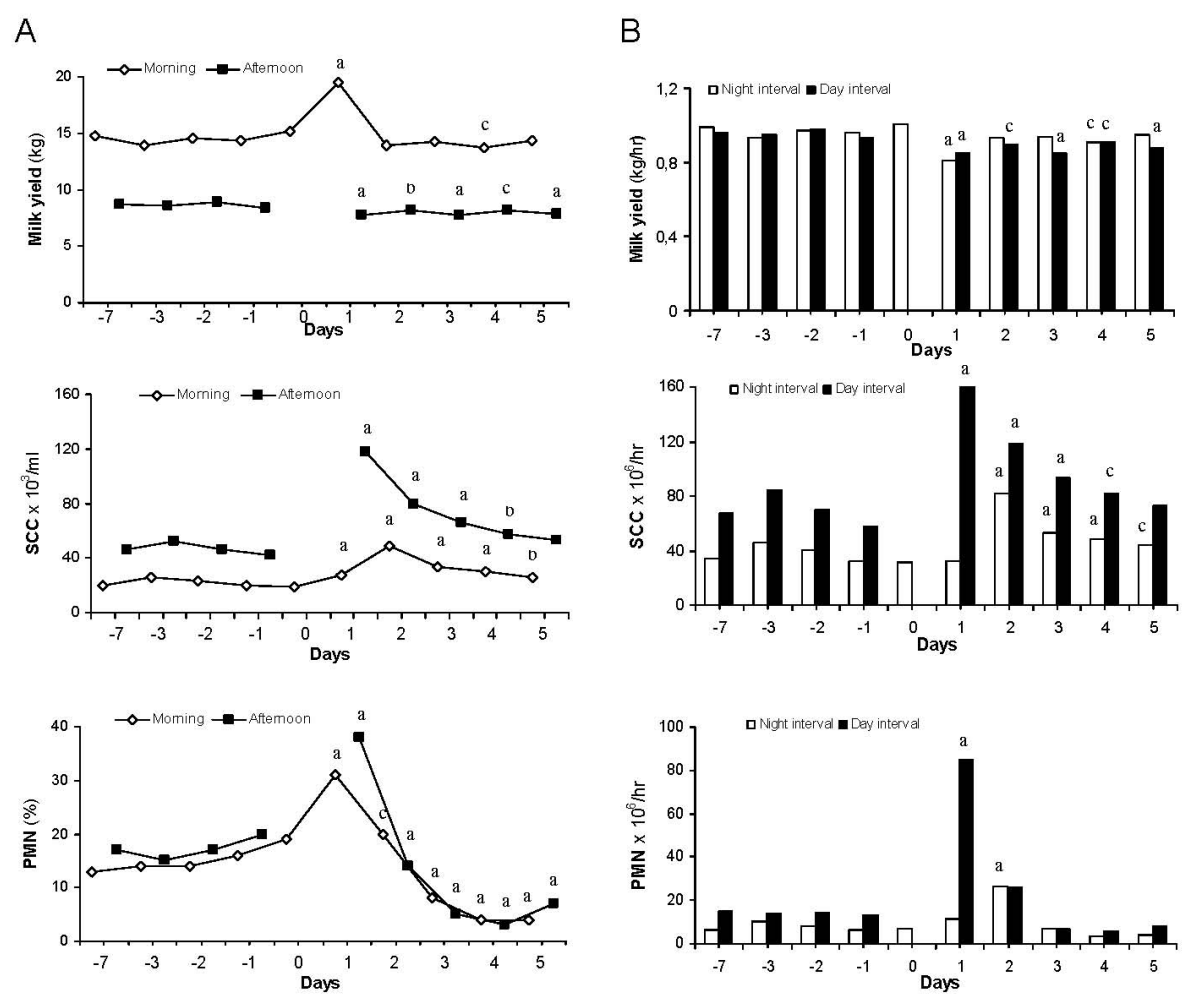
**Milk yield, somatic cell count (SCC) and concentration of polymorphonuclear leukocytes (PMN) in morning and afternoon milk (column A) and the calculated average output/hr during the interval between milkings (column B) before and after a single prolonged milking interval of 24 hrs**. The prolonged milking interval (PMI) occurred between the morning milkings day 0 and day 1. Night milking interval = the time between afternoon and morning milking. Day milking interval = the time between morning and afternoon milking. Data represent the LS-means. SE for yield, SCC and PMN in morning and afternoon milk was 0.59 and 0.39; 0.06 and 0.07 (10 logarithmic values); and 2.13 and 2.16, respectively. Letters indicate statistically significant differences between the sampling occasion and the baseline value before the PMI. a: p < 0.001, b: p < 0.01, c: p < 0.05.

**Figure 2:**
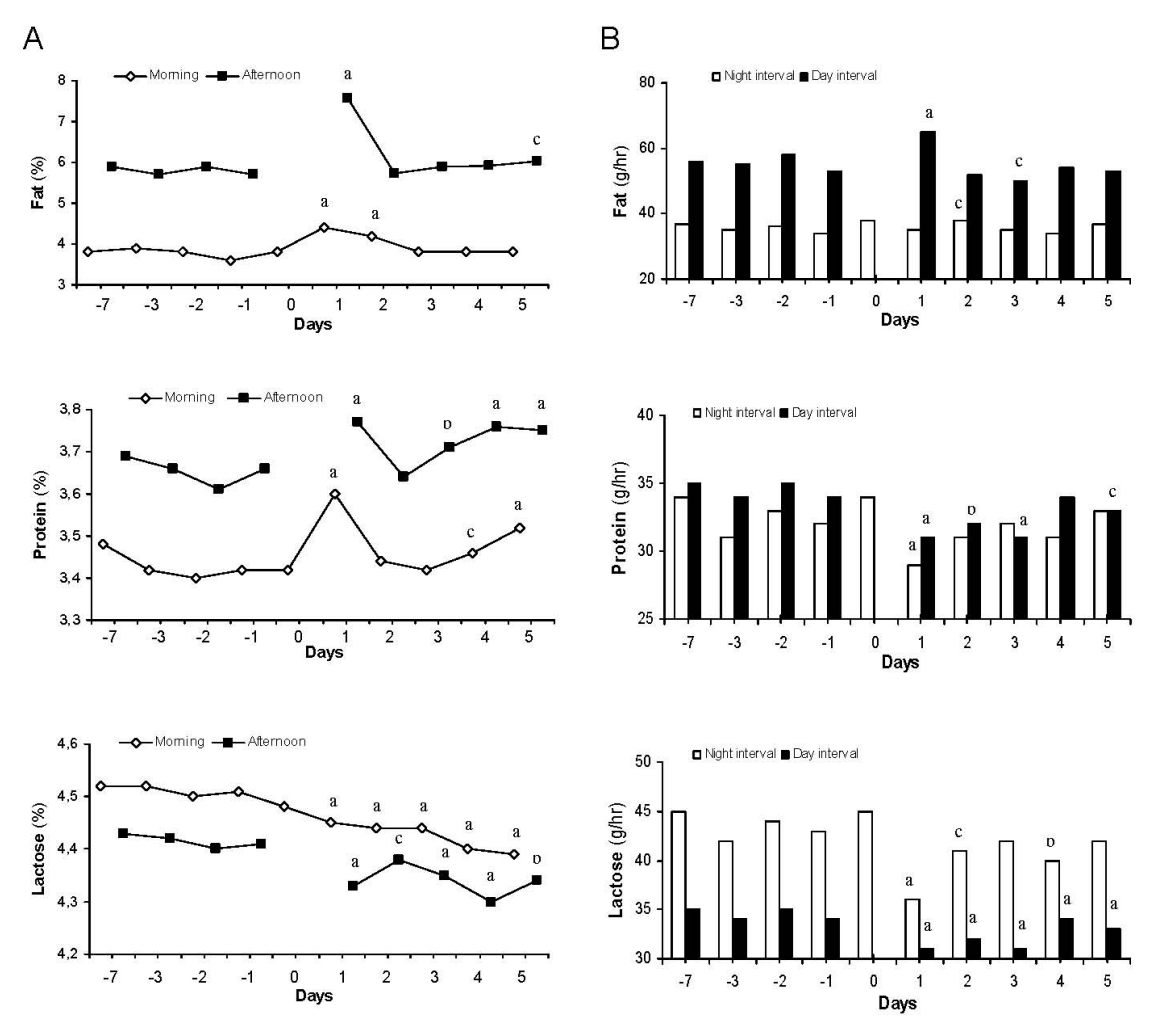
**The concentration of fat, protein and lactose in morning and afternoon milk (column A) and the calculated average output/hr during the interval between milkings (column B) before and after a single prolonged milking interval of 24 hrs**. The prolonged milking interval (PMI) occurred between the morning milkings day 0 and day 1. Night milking interval = the time between afternoon and morning milking. Day milking interval = the time between morning and afternoon milking. Data represent the LS-means. SE for fat, protein and lactose in morning and afternoon milk was 0.13 and 0.16; 0.06 and 0.05; and 0.03 and 0.03, respectively. Letters indicate statistically significant differences between the sampling occasion and the baseline value before the PMI. a: p < 0.001, b: p < 0.01, c: p < 0.05.

A total of 551 milk samples were collected and analyzed. Besides yield and concentrations (Fig. 1A and 2A), the output rate of each parameter during the time since the preceding milking is presented (Fig. 1B and 2B), except for casein, whey and FFA where total output/milking is used. The interval between morning and afternoon milking is denoted "day milking interval" (DMI) and that between afternoon and morning milking "night milking interval" (NMI). All values for each parameter were statistically compared within morning and afternoon milk, respectively, with the baseline value before the PMI.

### Milk yield

The baseline value of milk yield before the PMI was 14.6 kg at the morning milking and 8.6 kg in the afternoon. During the PMI milk yield accumulated in the udder and was significantly higher at the first morning milking (19.5 kg) day 1 while, on the contrary, it was significantly lower in afternoon milk (7.7 kg) compared to the baseline values, respectively (Fig. 1A). After day 1, morning milk yield returned to a level that was similar to the baseline value while afternoon milk yield remained significantly lower throughout the study.

The baseline value for yield *output *rate during the NMI and DMI, respectively, was 0,97 kg/hr and 0,95 kg/hr. It dropped significantly during the PMI and the subsequent milking interval day 1 (Fig 1B). Thereafter it varied but remained on a level that was significantly lower than the baseline for all DMI throughout the study but for NMI only at days 1 and 4.

### SCC

The SCC baseline value before the PMI was 21 × 10^3 ^for morning and 47 × 10^3 ^for afternoon milking, respectively. After the PMI, SCC increased significantly in both morning (the first milking after the PMI) and afternoon milking samples day 1 (Fig. 1A), to 27 × 10^3 ^and 118 × 10^3^respectively. This value represented the highest recorded SCC value in the afternoon milk while the peak in the morning milk was not observed until day 2 and with a notably lower magnitude than the peak in the afternoon milk day 1. After the peaks, the SCC in both morning and afternoon milk, declined but remained significantly above their baseline values, respectively, throughout the study.

The baseline value for *output rate *of somatic cells during the NMI and DMI, respectively, was 37 × 10^6 ^cells/hr and 70 × 10^6 ^cells/hr. The SCC output rate was in general lower during the NMI than during the DMI (Fig. 1B). The output during the PMI, was just slightly numerically increased (non-significantly) compared to the baseline value. After that the cell output rate increased dramatically and was approximately 5-fold increased during the subsequent milking interval day 1. The cell output rate thereafter declined but remained significantly increased above the baseline during both DMI and NMI until the last milking of the study.

### PMN

The leukocyte types observed in composite milk during the microscopic counting of PMN were PMN, monocyte-macrophages and lymphocytes. Only single epithelial cells were observed occasionally, which is in agreement with previous studies of milk with low SCC [23-25].

The baseline value of PMN before the PMI was 15% in morning and 17% in afternoon milk. After the PMI, the percentage of PMN was significantly increased day 1 in both morning and afternoon milk, 31% and 38%, respectively (Fig. 1A). It is noteworthy that the PMN peak in both morning and afternoon milk was observed during day 1, in contrast to the SCC which in morning milk did not peak until day 2. After the short and transient peak observed day 1, the percentage of PMN in milk started to decline day 2 and were statistically significantly *lower *than before the PMI from day 3 throughout the study in both morning and afternoon milk.

For PMN *output rate*, the baseline value during the NMI and DMI, respectively, was 7,5 × 10^6 ^cells/hr and 14 × 10^6 ^cells/hr. Similar to SCC, the output of PMN (Fig. 1B) was in general lower during the NMI than during the DMI. During the PMI the output rate of PMN increased with more than 50% compared to the baseline value, but the difference was not statistically significant (p = 0.11). Moreover, during each of the two subsequent milking intervals the increase of the output rate of PMN was huge compared to the baseline value (p < 0.001) for DMI and NMI, respectively. Thereafter, until the end of the study, the output rate of PMN declined to values that were numerically below the comparable baseline values, however statistically non-significant.

### Fat and FFA content

The fat baseline value before PMI was 3.8% at morning and 5.8% at afternoon milking, respectively. After the PMI, the fat percentage was significantly increased in both morning and afternoon milking samples day 1, (4.4% and 7.6%, respectively), and in morning milk day 2 (Fig. 2A). Thereafter the fat percentage declined and was not significantly changed in comparison with the baseline values, in either morning or afternoon milk, during the rest of the study.

The baseline *output rate *of fat during the NMI and DMI, respectively, was 36 g/hr and 56 g/hr. The output rate did not alter during the PMI but increased significantly during the two subsequent milking intervals day 1 and 2 (Fig. 2B). Thereafter it declined to values similar to the baseline values of DMI and NMI, respectively, except for occasionally during the DMI day 3.

FFA was analyzed only in afternoon milk. In contrast to all other milk constituents measured, no significant changes in the content of *FFA *(mEkv/l; SE = 0.08) were observed after the PMI although there was a numerical drop to day 1. However, the FFA content relative to the total fat content (mEkv/100 g of fat) decreased significantly (p < 0.001) from 1.76 before the PMI to 1.33 day 1. When data only from the cows that showed a particularly pronounced SCC peak day 1 (n = 9; SE 0.10) was analyzed a similar significant drop in the FFA content relative to the total fat content from 2.05 before the PMI to 1.52 day 1 (p = 0.01) was observed and additionally the value at day 5 was lower (p < 0.05) than before the PMI.

### Protein, casein and whey protein content

The baseline value of *protein *content was 3.43% for morning and 3.66% for afternoon milking. After the PMI, the protein content increased significantly in both morning and afternoon samples to 3.60% and 3.77%, respectively, day 1 (Fig. 2A). After the pronounced peak, values rapidly declined and already day 2, the protein percentage was not significantly different compared to that before the PMI in either morning or afternoon milk, respectively. However, a significant increase was recorded again in the afternoon milking day 3 and the protein percentage thereafter remained significantly increased in both morning and afternoon milk, respectively, during the rest of the study.

The baseline value for *output rate *of protein during the NMI and DMI, respectively, was 33 g/hr and 35 g/hr. The output rate decreased significantly during the PMI and the subsequent milking interval, day 1. The output during the DMI remained significantly decreased throughout the study (except for day 4). Numerically, the output rate was lower also during the NMI compared to the baseline, but statistically non-significant.

Casein and whey were analyzed only in afternoon milk. The average *casein *percentage (SE = 0.04) increased significantly after the PMI from 2.66% day -1 to 2.73% day 1 (p < 0.01) while the total output per milking decreased from 221.6 g day -1 to 207.7 g day 1 (p < 0.05). The *whey protein *percentage, accordingly, decreased significantly from 1.08% day -1 to 1.04% day 1 (p < 0.01; SE = 0.02). The total whey output per milking was not significantly changed (p = 0.08) although there was a numerical decrease from day -1 (90.2 g) to day 1 (79.6 g). When data only from the cows that showed a particularly pronounced SCC peak day 1 (n = 9) was analyzed, no significant changes in either casein percentage (SE = 0.05) or total output, or in whey percentage (SE = 0.03) were observed. However, the total output of whey protein per milking was decreased from 102.0 g day -1 to 83.2 g day 1 (p < 0.05).

### Lactose

The baseline of lactose before PMI was 4.51% for morning and 4.41% for afternoon milk. After the PMI, the lactose content dropped significantly in both morning and afternoon milk to 4.45% and 4.33%, respectively, at day 1 (Fig. 2A). The lactose concentration remained significantly decreased in morning as well as afternoon milk compared to the baseline values, respectively, throughout the study.

The baseline of *output rate *of lactose during the NMI and DMI, respectively, was 44 g/hr and 35 g/hr. The rate of lactose output decreased significantly during the PMI and the subsequent milking interval day 1 compared to the baseline values, respectively, and remained significantly decreased during the DMI for the rest of the study. In the NMI the output rate of lactose also remained at a numerically lower level, but significantly lower only in day 2 and day 4.

## Discussion

The main findings of the present study were that *during *the PMI there was a distinct increase in proportion of PMN while the increase in SCC was slight and that the most pronounced changes were observed in the *second milking after *the PMI, in the afternoon day 1. In *morning *milk the peak SCC value did not occur until day 2 and, unexpectedly, concomitantly with a *decrease *in the proportion of PMN. The alterations in milk composition were numerically slight with lowered relative FFA content and did not show any adverse influence on the milk quality.

The rise in SCC observed after the PMI is in principal in accordance with previous studies [3,12] although information on milk SCC after a *single *PMI is scarce. The peak value in morning milk, in contrast to afternoon milk, was not observed until day 2. The SCC is, in general, known to be lower in morning milk than in afternoon milk [26]. It has been ascribed to a different degree of dilution of the cells by the different milk volumes at the two daily milking occasions due to uneven milking intervals. This could partly have explained the SCC results also in the present study since the accumulated milk volume during the PMI was notably high in the first morning milking thereafter. The changes in SCC during and after the PMI were, however, in accordance with the number of cells entering the milk *per time unit *(Fig. 1B), a measure which exclude the effect of dilution. Why the highest SCC value and distinctly increased recruitment/hr of total somatic cells to the milk, was not observed until the afternoon milking day 1 while sharply increased PMN percentage in milk was observed already in the morning, remains to be explained. Early increase in the proportion of milk PMN has also been reported by others, however, concomitantly with increased SCC [3].

Which factors that might have triggered the PMN migration can be discussed. It could be argued that the large accumulated milk volume during the PMI and extension of the udder might have caused increased permeability with subsequent leakage of inflammatory mediators into milk from blood. Stelwagen et al. [27] described a temporary, reversible disruption of tight junction (TJ) integrity, due to increased intramammary pressure by milk accumulation after a 24-h milking interval. They discussed whether this could facilitate the migration of leukocytes into the mammary gland and explain the increased SCC observed after a single PMI in some studies [3,12]. We observed *decreased *concentration of serum proteins (whey proteins) in the milk after the PMI, which indicates that the mammary endothelial and epithelial permeability was not increased in this study. The recruitment of PMN was further enhanced during day 1 when the udder was emptied twice and not extended, which speaks for that the PMN migration was influenced by factors not related to a large milk volume and accumulation of milk, per se.

It is noteworthy that in several respects the proportion of PMN appeared not to follow the SCC in the way that has previously been shown in cow milk under various inflammatory and physiological conditions [7,9-11]. Although the increase in SCC *during *the PMI was slight (from 21 × 10^3 ^to 27 × 10^3^), the proportion of PMN was doubled (from 15% to 31%). Further, when the most pronounced SCC increase in morning milk day 2 was observed, the percentage of PMN had sharply *declined*. Thus, this increase in SCC was, apparently, mainly attributable to other kinds of leukocytes. At this time, the proportion of PMN also switched to become higher in morning than in afternoon milk in contrast to the SCC and what is usually seen [26]. The results speak for a special background to the recruitment of leukocytes to milk after a PMI and to the increased proportion of PMN. It is further supported by the extremely rapid return of the proportion of PMN to the baseline level. Manlongat et al. [28] identified the presence of "physiological" chemotactic factors in mammary secretions influencing the recruitment of PMN to goat's milk in late lactation and emphasized that increased infiltration of PMN to the mammary gland under certain circumstances must not necessarily be a result of a pathological process. They also observed different activity of specifically mononuclear leukocyte chemoattractants during the lactation period. The results from the current study are in accordance with the findings by Manlongat et al. [28] and suggest the presence of physiological chemotactic factors in cow milk active in response to a long milking interval. This remains to be further explored by specifically examining the immunophysiological background to the SCC peaks which was not the aim of the present study.

Another interesting finding in the present study is that the proportion of PMN decreased to values that were below the baseline value in both morning and afternoon milk from day 2 and throughout the study. In contrast, the SCC after declining still remained above the baseline value. These results were highly significant even if the changes were numerically modest and indicate a relative decreased attraction of PMN to the milk during several days after the PMI, in favour of recruitment of mononuclear leukocytes.

The milk composition was significantly changed the first day after the PMI but the changes in the following days were not consistent except for *lactose *that was lower throughout the study. Lactose is the key for osmotic regulation in the udder and a drop in lactose content is often accompanied by a drop in milk yield. Either it could be ascribed to leakage out of lactose from the milk through impaired TJs or it could be attributed to lower synthesis of lactose during the PMI. Since the unchanged, or even slightly decreased, content of serum proteins in milk speaks for an unaffected integrity of the TJs, a lower synthesis is more likely. A plausible background to a lowered synthesis of lactose after the PMI is a decreased content of α-lactalbumin. It is well known that this protein is a coenzyme in the synthesis of lactose. α-lactalbumin constitutes almost 20% of the serum proteins (whey proteins) [29] which were shown to decrease significantly in the present study. Since lactose plays a key role in regulating the osmotic pressure it is also influenced by the content of ions in milk. However, in the present study no analyses were done on the content of sodium and potassium ions.

The elevated *protein content *was apparently due to an increase in *casein *content while the content of serum proteins decreased. Increased casein content has been reported from previous studies of PMIs when applying once-daily milking, regularly. Claesson [17] observed a higher concentration of casein during once-daily than twice-daily milking as did also Lacy-Hulbert et al. [30]. The increase has been explained by the large size of the casein micelles, making them un-capable of leaking out through TJs to the blood compartment. In the present study the increased proportion of casein was probably, at least partly, attributable to a concentration effect by the decreased afternoon milk yield at day 1. This is further supported by the observation that the total output of casein decreased. The decreased casein output could also be an effect of increased presence of plasma proteolytic enzymes. In earlier studies it has been observed that short milking intervals are related to lower plasmin activity in the milk compared to milk obtained after a long milking interval [31,32]. The decreased serum protein content in milk observed indicates that the permeability of endothelium and epithelium was not increased in the present study making increased plasmin activity in milk after the PMI less likely.

The *fat *content was significantly increased after PMI. The changes in fat might at least partially be ascribed to a concentration effect. FFA is undesirable in milk due to its degradation of fat quality and rancid flavour. Accumulation of FFA in the milk is related to higher hydrolysis of triglycerides catalyzed by lipoprotein lipase. It is known from previous studies that short and irregular milking intervals may result in elevated FFA content in milk [33,34]. In the current study, the level of FFA/100 g fat decreased significantly. Since fat content increased while the FFA decreased, apparently, there was no effect of lipoprotein lipase, originating from blood plasma. This further supports that the TJs kept their integrity during the study.

As expected, the PMI resulted in significantly elevated milk *yield *at the first milking in the morning of day 1 due to accumulation of milk in the udder. In the rest of the study the *morning *milk yield was not changed compared to before the PMI. In contrast to the morning milk, the *afternoon *milk yield day 1 was reduced and, remarkably, remained significantly lower than the baseline value throughout the study. A probable mechanism behind that afternoon yield particularly was reduced is difficult to identify. There is little information available of the effect of a single PMI on the milk yield, but decreased yield has been reported previously [12]. A considerable reduction of milk yield was also observed by Claesson et al. [35] when one milking per week was omitted. In several studies a lowered milk yield has been observed in cows when milking frequency was reduced to once per day [14,27,36]. These results show the long term effect of less frequent milking and regularly higher intramammary pressure. In the present investigation the pressure was increased only occasionally during a short period of time. The significantly decreased lactose content observed might still indicate that the drop in yield could be due to a negative influence of high intramammary pressure on the milk secreting cells. However, Stelwagen and Lacy-Hulbert [3] suggested that a single PMI did not cause any damage to the mammary secretory epithelium. Additionally, a lingering effect of the increased pressure during the PMI over several days while the milking continued twice per day seems not to be a plausible explanation for the lowered milk yield. It still remains to be explained.

## Conclusion

The results from the present study indicate that there might be a special chemotactic background to the increased proportion of PMN in milk, observed without any obvious inflammatory challenge, during and after the PMI. The recruitment of PMN was further enhanced the first day after the PMI when the udder was milked twice daily. This speaks for that the PMN migration was influenced by factors not related to a large milk volume and accumulation of milk, per se. Milk composition was not markedly changed after the PMI, except for lactose, but it did not influence milk quality. Several findings indicate that the PMI did not affect the TJs.

## Competing interests

The authors declare that they have no competing interests.

## Authors' contributions

BL carried out the practical work, performed neutrophil counting, compiled the results, participated in the statistical analysis and interpretation of results, drafted the manuscript and participated in its revision.

KÖ and KSS designed the study. KÖ supervised and participated in the practical work and neutrophil counting, assisted in interpretation of the results and was main responsible for supporting the drafting and for revision of the manuscript. KSS assisted in interpretation of the results and helped to draft and revise the manuscript. EW participated in the practical work of the study, was main responsible for performing the statistical analyses, helped in interpreting the results and revising the manuscript. All authors read and approved the final manuscript.
